# Accessing the public MIMIC-II intensive care relational database for clinical research

**DOI:** 10.1186/1472-6947-13-9

**Published:** 2013-01-10

**Authors:** Daniel J Scott, Joon Lee, Ikaro Silva, Shinhyuk Park, George B Moody, Leo A Celi, Roger G Mark

**Affiliations:** 1 Harvard-MIT Division of Health Sciences and Technology, Cambridge, MA 02139, USA; 2 School of Public Health and Health Systems, University of Waterloo, Waterloo, Ontario N2L 3G1, Canada

**Keywords:** MIMIC-II, Relational database, Intensive care, Clinical research, SQL, Virtual machine

## Abstract

**Background:**

The Multiparameter Intelligent Monitoring in Intensive Care II (MIMIC-II) database is a free, public resource for intensive care research. The database was officially released in 2006, and has attracted a growing number of researchers in academia and industry. We present the two major software tools that facilitate accessing the relational database: the web-based QueryBuilder and a downloadable virtual machine (VM) image.

**Results:**

QueryBuilder and the MIMIC-II VM have been developed successfully and are freely available to MIMIC-II users. Simple example SQL queries and the resulting data are presented. Clinical studies pertaining to acute kidney injury and prediction of fluid requirements in the intensive care unit are shown as typical examples of research performed with MIMIC-II. In addition, MIMIC-II has also provided data for annual PhysioNet/Computing in Cardiology Challenges, including the 2012 Challenge “Predicting mortality of ICU Patients”.

**Conclusions:**

QueryBuilder is a web-based tool that provides easy access to MIMIC-II. For more computationally intensive queries, one can locally install a complete copy of MIMIC-II in a VM. Both publicly available tools provide the MIMIC-II research community with convenient querying interfaces and complement the value of the MIMIC-II relational database.

## Background

The Multiparameter Intelligent Monitoring in Intensive Care II (MIMIC-II) database [1] (http://physionet.org/mimic2) is a public research archive of data collected from patients in intensive care units (ICUs). Although other clinical research databases exist [2,3], such databases are often privately owned, have highly restricted access or require fees for access. MIMIC-II has been fully deidentified in a Health Insurance Portability and Accountability Act (HIPAA) compliant manner and is available free of charge for public use, subject to completion of an appropriate online human-subjects training course and signing of a data use agreement. The database is available via PhysioNet [4,5], a web-based resource for the study of physiologic data.

The data comprising MIMIC-II was collected at the Beth Israel Deaconess Medical Center in Boston, MA, USA from patients who were admitted from 2001 to 2008. The available clinical information includes: patient demographics, laboratory test results, vital sign recordings, fluid and medication records, charted parameters and free-text reports such as nursing notes, imaging reports and discharge summaries. There is a second component of MIMIC-II consisting of high resolution waveform recordings of electrocardiograms, blood pressures, pulse plethysmograms and other monitored signals that were archived from bedside monitors for a subset of the patients. The waveforms, and derived trends and alarms are the subject of much research interest [6-8]. Here, however, we focus primarily on the “clinical data”, stored in a relational database. For a detailed description of the MIMIC-II database, please see [1]. The MIMIC-II project was approved by the Institutional Review Boards of the Beth Israel Deaconess Medical Center and the Massachusetts Institute of Technology (Cambridge, MA, USA). The requirement for individual patient consent was waived because clinical care was not affected and all protected health information (PHI) was deidentified.

As of the end of 2012, over 500 users have been approved for access to the MIMIC-II relational database, which reflects researchers’ interest in the clinical data of MIMIC-II. Numerous innovative and significant studies on a broad range of topics are based on MIMIC II and establish its importance. The software tools that make it feasible for a large worldwide community of investigators to draw on MIMIC II are essential contributors to its value and utility for intensive care research. Providing public access to a relational database for users who are geographically separated and from a wide range of backgrounds is a challenging task. While there are tools available for web-based administration such as phpPgAdmin [9] and even searching of clinical data [10], they are not always appropriate in any given situation. For MIMIC-II, we have developed an easy-to-use, read-only interface capable of performing exploratory searches and a more powerful tool for complex data processing. These two access tools currently serve as the main gateways to the MIMIC-II relational database. In the present article, we describe their implementations and vital roles in conducting clinical research using MIMIC-II.

## Implementation

The MIMIC-II relational database (version 2.6) contains records from over 32,000 subjects, including over 7,000 neonatal patients. The raw data is stored in various base tables, generally organized by subject, hospital and ICU-stay IDs. Several database views, which summarize and collate information, have been generated to allow users to become familiar with the available data and to find records of interest. Users can access the database via a web-based online tool (QueryBuilder) and a downloadable virtual machine (VM) image, which are discussed in the ensuing sections. Flat file exports of the database tables and a PostgreSQL compatible dump file are also available, but are not discussed here. To the best of our knowledge, there are no other publicly available software tools that allow users to query a clinical database in SQL (Structured Query Language), either in a web-browser or in a virtual machine environment.

### QueryBuilder web-based tool

**Figure 1:**
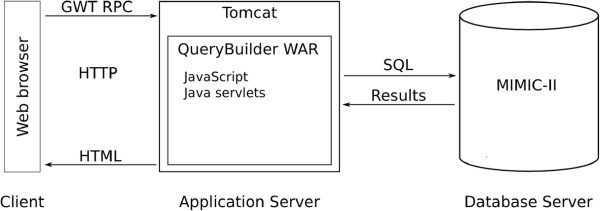
**QueryBuilder system infrastructure.** The user connects to QueryBuilder using a web browser. When the user submits an SQL query, it is transmitted to the application server and executed on the database server via JDBC. The query results are returned to the user via the application server and displayed.

QueryBuilder is a web-based database query tool developed using the Google Web Toolkit (GWT) [11] and ExtGWT widget library [12]. Figure 1 shows the system infrastructure. The QueryBuilder application is hosted on a Tomcat 7 application server and connects to an Oracle 11g database containing clinical data from MIMIC-II. Queries are submitted to the application server using a GWT Remote Procedure Call (RPC) and are executed in the database using Java DataBase Connectivity (JDBC) [13]. The results are passed back through the application server to the user’s web browser.

The GWT framework allows developers to write code in Java, which is then compiled into a highly optimized browser-independent JavaScript web application. GWT provides a “development mode” in which the Java code is dynamically translated to JavaScript and displayed using a browser plugin. For production systems, GWT builds a Web application ARchive (WAR) file containing optimized JavaScript that works across a wide range of browsers and platforms. The WAR contains Java servlets for server side processing and can be deployed on any standard application server.

QueryBuilder, which is accessible through desktop or mobile web browsers, allows users to explore the structure of the various tables and views in the database and to examine the relationships among them. SQL queries allow users to examine and process the data as desired; the resulting datasets can be exported in CSV (Comma-Separated Values) format for further processing. In order to prevent a given user from excessively consuming shared resources on QueryBuilder (e.g., exporting all tables in MIMIC-II), we limited the maximum number of exportable rows to 1,000.

### Virtual machine

The increasing number of users, and their desire to run more complex queries, has begun to overload the computer systems hosting QueryBuilder. To mitigate this problem, we have developed a system allowing users to run a copy of the relational database on their own computers, providing much faster, uncongested access using a VM. A VM is a completely isolated operating system installation that can be run within a host environment. The MIMIC-II VM employs Oracle’s VirtualBox virtualization environment, providing an Ubuntu 10.04 Linux operating system distribution and a pre-configured PostgreSQL 8.4 database server.

To use the MIMIC-II VM, users must first install the VirtualBox host software, and download the MIMIC-II VM image for import into VirtualBox. Once the VM has been started, a simple script will download and import the MIMIC-II database into the local PostgreSQL server. The resulting system contains a complete clone of the MIMIC-II relational database that can be queried using a command line client, a GUI (Graphical User Interface) desktop application (pgAdmin III), and JDBC interfaces. The VM also includes an SQL cookbook which is a compilation of example SQL queries that users can use as a starting point for their research studies.

We also created a demo VM (and, to suit users’ preferences, a bootable ISO image) containing data from 4,000 patients who have been deceased for two years or more. Since the demo VM and the ISO image contain neither PHI, nor free text, nor any data from recently living individuals, they are exempt from HIPAA restrictions, and interested researchers may download them freely.

## Results

**Figure 2:**
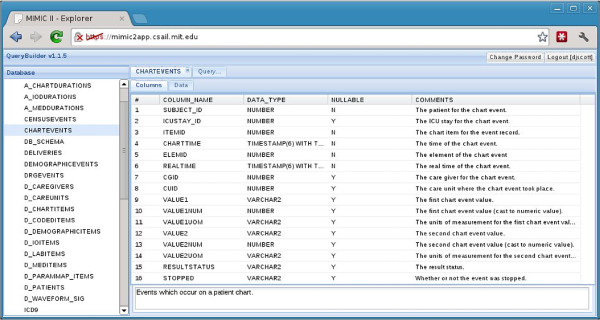
**QueryBuilder screenshot.** A screenshot of QueryBuilder, showing information about the CHARTEVENTS table in MIMIC-II.

**Figure 3:**
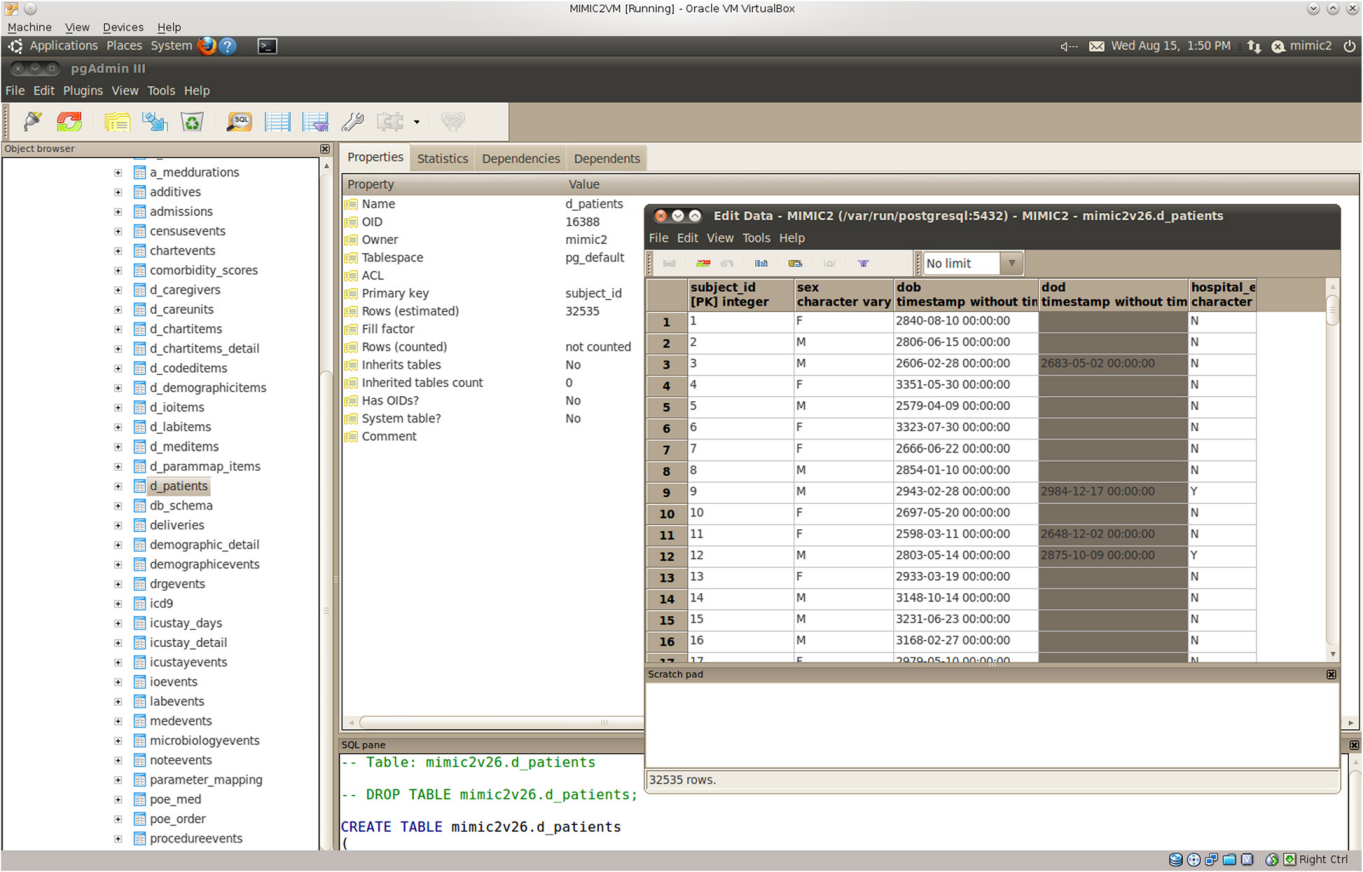
**MIMIC-II VM screenshot.** A screenshot of the MIMIC-II virtual machine, showing the D_PATIENTS table in pgAdmin.

Both QueryBuilder and the MIMIC-II VM are currently available to MIMIC-II users free of charge. A link to QueryBuilder, the downloadable VM image, as well as related documentation and instructions for gaining access are available on PhysioNet (http://physionet.org/mimic2). Figures 2 and 3 are screenshots of QueryBuilder and the VM, respectively.

Both QueryBuilder and the VM are routinely used by researchers around the world. As of November 6, 2012, 508 MIMIC-II users have a QueryBuilder account. Between January 1 and November 7, 2012, an average of 4.5 users logged into QueryBuilder per day and a total of 221 unique users logged in at least once. The VM with complete data have been downloaded 129 times between July 2011 and October 2012, whereas the demo VM and bootable image have been downloaded 2,234 times between August 2011 and October 2012.

Although QueryBuilder and the VM provide immediate access to the MIMIC-II relational database, the user needs to have working knowledge in both SQL and the MIMIC-II database schema. SQL is a rare skill among clinicians, and becoming familiar with the structure of the MIMIC-II clinical data requires substantial time and effort. In order to guide new MIMIC-II users, we present a few example queries and research studies in the subsequent sections. We recommend using QueryBuilder for the simple example queries in Section “Example usage” and using the VM for the computationally expensive studies in Section “Applications”.

### Example usage

The ICUSTAY_DETAIL view summarizes ICU stays for all patients and can be used to obtain general statistics for the entire population of MIMIC-II. The following example query obtains ICU mortality statistics broken down by gender.

**SELECT** gender, icustay_expire_flg, **COUNT**(*) **FROM** mimic2v26.icustay_detail **WHERE** subject_icustay_total_num = 1 **GROUP BY** gender, icustay_expire_flg

The results from this query are shown in Table 1, which indicates that among patients with only one ICU stay in the database, there are more males than females, and that males have a lower ICU mortality rate (6.2%) than females (7.2%). One can obtain hospital mortality by querying the hospital_expire_flg.

**Table 1:** ICU mortality statistics

**gender**	**icustay_expire_flg**	count (*)
M	Y	958
M	N	14393
F	Y	876
F	N	11207
(null)	Y	2
(null)	N	43

ICU mortality statistics generated by executing the example query. There are some people for whom gender was not recorded, indicated by ‘(null)’.

The ICUSTAY_DETAIL table is also used to obtain patient cohorts by using the WHERE clause to restrict the query to obtain ICU stays of interest. The second example query obtains all ICU stays for patients who have a SAPS (Simplified Acute Physiology Score) I [14] score between 15 and 20, are between 20 and 30 years old, had 2 ICU admissions in total and died in the hospital.

**SELECT** icustay_id, subject_id, gender, dob, dod **FROM** mimic2v26.icustay_detail **WHERE** icustay_admit_age **BETWEEN** 20 **AND** 30 **AND** sapsi_first **BETWEEN** 15 **AND** 20 **AND** subject_icustay_total_num = 2 **AND** hospital_expire_flg = ‘Y’

The query returns three rows as shown in Table 2 (for patient privacy, an offset, randomly chosen for each patient individually, has been added to all dates in the original data to obtain surrogate dates). Despite its apparently simple constraints, the query is actually quite specific, and there are only three subjects, all male, from over 26,000 adults who meet the criteria. Furthermore, although the query sought patients with two ICU stays, the results show only one stay for each patient. This is for two possible reasons: 

1. The patient’s SAPS I score during his other ICU admission was not between 15 and 20.

**Table 2:** Specific patient cohort

**icustay_id**	**subject_id**	**gender**	**dob**	**dod**
5995	4828	M	3055-10-23	3081-09-03
30325	24431	M	2702-02-11	2726-09-29
41166	27109	M	3032-11-04	3061-07-09

Results of the example query in the text. ICUSTAY_ID and SUBJECT_ID identify the ICU stay and the patient; DOB and DOD are surrogate dates of birth and death; see text for discussion.

2. The patient’s age was not between 20 and 30 for one of his two ICU admissions.

We can obtain all of the ICU stays for the subjects who were returned in Table 2 by querying for specific subject IDs.

**SELECT** icustay_id, subject_id, icustay_admit_age, sapsi_first, icustay_intime, icustay_outtime **FROM** mimic2v26.icustay_detail **WHERE** subject_id **IN** (4828,24431,27109)

The results in Table 3 show that each patient did have two ICU stays, but his SAPS I score was available for only one of them. The records not listed in Table 2 failed to meet the criteria of the previous query, most likely due to missing data for one or more parameters needed to calculate the SAPS I score.

**Table 3:** Patient data

**icustay_id**	**subject_id**	**icustay_admit_age**	**sapsi_first**	**icustay_intime**	**icustay_outtime**
5994	4828	25.19514	(null)	3081-01-02 14:13:00 -05:00	3081-01-02 21:39:00 -05:00
5995	4828	25.85458	15	3081-08-30 21:41:00 -05:00	3081-09-03 04:09:00 -05:00
30325	24431	24.61293	17	2726-09-22 00:17:00 -05:00	2726-09-29 20:58:00 -05:00
30326	24431	24.64457	(null)	2726-10-02 18:44:00 -05:00	2726-10-02 18:51:00 -05:00
41166	27109	28.6202	18	3061-06-17 17:07:00 -05:00	3061-06-20 13:43:00 -05:00
41167	27109	28.63751	(null)	3061-06-24 03:44:00 -05:00	3061-07-09 21:51:00 -05:00

The data shows that the SAPSI_FIRST column is ‘null’ in three rows, explaining why these rows were not returned in Table 2. The missing SAPS I scores are most likely due to missing data for the parameters used to calculate the score.

The MIMIC-II database contains complex, detailed data and apparently simple queries can return unexpected results. The rich, detailed information it contains has stimulated a variety of research interests.

### Applications

MIMIC-II has attracted research in data mining, pattern recognition and signal processing. There have been a wide variety of publications based on the data contained within MIMIC-II and its public availability encourages reproducible research and permits comparison of results. We now discuss two of the recent research problems that have been investigated using data from MIMIC-II. Subsequently, the PhysioNet/Computing in Cardiology (CinC) Challenges that utilized MIMIC-II are also described. The examples below illustrate what kinds of clinical research are possible with the MIMIC-II relational database.

#### Acute kidney injury

Acute kidney injury (AKI) is a serious and frequent condition in critically ill patients [15]. There are established criteria [16] defining three severities of AKI based on patient urine output over 6, 12 or 24 hour periods and increases in serum creatinine levels over a two-day window. MIMIC-II contains hourly urine output measurements and daily serum creatinine laboratory test results that permit a thorough investigation into the AKI classifications. Using the data in MIMIC-II, we were able to determine AKI stages for all patients and build multivariate logistic regression models to determine whether AKI stages can be used as biomarkers of increased hospital mortality [17]. Owing to the high temporal resolution of the data, we were able to build models for a large range of urine output thresholds and durations to determine that the existing AKIN definitions employ clinically meaningful criteria [18].

#### Prediction of fluid requirement in the ICU

The first 72 hours after admission are critical for ICU patients. Suboptimal fluid management during this period can result in episodes of hypotension, leading to reduced organ perfusion. In practice, clinicians perform the difficult task of estimating maintenance fluid requirement by estimating fluid loss. Providing an accurate prediction of a patient’s fluid requirements would assist clinicians in making their decision.

MIMIC-II contains detailed fluid input/output measurements as well as vasopressor administration, demographics and physiologic variables. Using data from the first day of a patient’s ICU admission in a linear regression model combined with a Bayesian network, Celi *et al.* were able to accurately estimate patient fluid requirements for day two^a^[19].

#### PhysioNet/Computing in cardiology challenges

The annual PhysioNet/CinC Challenges (http://www.physionet.org/challenge/) invite participants to tackle clinically interesting problems. The challenges in 2009 [20] to predict hypotensive episodes in the ICU and 2010 [21] to attempt to reconstruct missing or corrupted signals, both used data from the MIMIC-II database. The 2012 PhysioNet challenge entitled “Predicting Mortality of ICU Patients” also used MIMIC-II data and asked participants to develop a patient-specific prediction of in-hospital mortality. The dataset consisted of MIMIC-II records from 12,000 ICU stays each at least 48 hours in duration providing up to 41 different variables. Five of the 41 were “general descriptors” (recordID, age, gender height and weight), recorded once, on admission. The remainder were “time series” variables such as vital signs and laboratory test results and were recorded multiple times throughout the 48 hour period. The aim of the challenge was to predict for each patient, whether they died in the hospital. Participants discussed their approaches to the challenge problem during the CinC 2012 conference (http://physionet.org/challenge/2012/).

## Discussion

The MIMIC-II database is a valuable research tool that is gaining popularity as it is expanded and improved over time. Its clinical data can be accessed using a variety of methods, including the web-based QueryBuilder and standalone virtual machine technology. These publicly available software tools play a vital role in connecting a broad community of researchers to MIMIC-II, providing them with immediate access to a one-of-a-kind ICU database and making it feasible for them to perform a wide variety of innovative studies with it.

Typically, a new MIMIC-II user would utilize QueryBuilder and the VM to conduct a clinical study in the following steps: 

1. Explore the clinical data in MIMIC-II using the demo VM and conduct a feasibility test for an envisioned research study.

2. Use QueryBuilder to conduct a further feasibility test by looking in the tables that are not part of the demo VM and by checking cohort size.

3. Write and debug an appropriate SQL query in QueryBuilder to extract desired patient data.

4. If the final SQL query requires substantial computing time or the results contain more than 1,000 rows, run the query in the VM with complete data.

In our experience, clinical research such as that presented in this article is best approached using an inter-disciplinary team combining clinicians who provide the research direction and interpretation of results with engineers who provide data extraction and statistical modeling [22].

Being a web-based tool, QueryBuilder ensures minimal setup time and effort. MIMIC-II users only need a web browser and Internet connection to be able to launch QueryBuilder. Installing a complete MIMIC-II VM on a local computer involves more steps and requires a longer time, but is an effective method when the shared resources for QueryBuilder become the bottleneck in conducting a research study.

We are working to introduce and improve tools for searching and visualizing the data available in MIMIC-II. Our existing QueryBuilder and VM require users to know or to learn SQL; our next generation of tools will provide an intuitive graphical interface that will be immediately accessible to a wider user community that includes many more clinicians. Additionally, we are expanding the database by adding additional patient records, and enlarging the records of existing patients. Improved tools and expansion of the database will further support retrospective clinical research.

In the present article, we have discussed simple example SQL queries as well as representative clinical studies that have been performed using MIMIC-II. We have also described the PhysioNet/CinC 2012 Challenge “Predicting Mortality of ICU Patients”. These examples hint at the range of problems that can be studied using MIMIC-II. They illustrate how investigators can formulate and answer research questions using open-source tools to explore the rich contents of the first (and so far the only) large and publicly available database for intensive care research.

## Conclusions

MIMIC-II is an invaluable public database for intensive care research, and we have successfully developed two freely available tools that facilitate accessing MIMIC-II. QueryBuilder is a web-based tool that allows a user to query MIMIC-II in SQL. For more computationally intensive queries, one can locally install a complete copy of MIMIC-II in a VM. A demo VM is also available for interested users who wish to explore MIMIC-II with minimal setup time. We believe that QueryBuilder and the MIMIC-II VM are integral parts of the MIMIC-II research community, which is corroborated by extensive utilization of both tools by MIMIC-II users.

## Availability and requirements

• Project name: QueryBuilder and MIMIC-II virtual machine

• Project home page: http://physionet.org/mimic2

• Operating system(s): Platform independent

• Programming language: Java, SQL

• Other requirements: Any web browser, Oracle VirtualBox

• License: Open source

• Any restrictions to use by non-academics: None

## Endnote

^a^The provided accuracy was 77.8%, which is the percentage of correctly estimated fluid requirements when the actual fluid requirements in the test dataset were divided into quartiles.

## Competing interests

The authors declare that they have no competing interests.

## Authors’ contributions

DS developed QueryBuilder and the MIMIC-II VM, and also wrote most of the manuscript. JL wrote parts of the manuscript and helped formulate the SQL query examples and select example research studies. LC and RM conducted the featured research studies that utilized MIMIC-II and also contributed to the query examples. IS and SP developed the MIMIC-II VM. GM distributed MIMIC-II, QueryBuilder, and the VM via PhysioNet. All authors critically revised the manuscript. All authors read and approved the final manuscript.

## Pre-publication history

The pre-publication history for this paper can be accessed here:

http://www.biomedcentral.com/1472-6947/13/9/prepub

## References

[B1] Saeed M Villarroel M Reisner AT Clifford G Lehman L Moody G Heldt T Kyaw TH Moody B Mark RG Multiparameter intelligent monitoring in intensive care II (MIMIC-II): A public-access intensive care unit database Crit Care Med 2011 39 5 952 960 10.1097/CCM.0b013e31820a92c6 21283005 PMC3124312

[B2] Lowe HJ Ferris TA Hernandez PM Weber SC STRIDE–An integrated standards-based translational research informatics platform AMIA Annu Symp Proc 2009 2009 391 395 [http://view.ncbi.nlm.nih.gov/pubmed/20351886] 20351886 PMC2815452

[B3] Stow PJ Hart GK Higlett T George C Herkes R McWilliam D Bellomo R Development and implementation of a high-quality clinical database: the Australian and New Zealand intensive care society adult patient database J Crit Care 2006 21 2 133 141 [http://www.sciencedirect.com/science/article/pii/S088394410500198X] 10.1016/j.jcrc.2005.11.010 16769456

[B4] Goldberger AL Amaral LAN Glass L Hausdorff JM Ivanov PC Mark RG Mietus JE Moody GB Peng CK Stanley HE PhysioBank, PhysioToolkit, and Physionet: Components of a new research resource for complex physiologic signals Circulations 2000 101 23 e215 e220 [http://www.physionet.org] 10.1161/01.CIR.101.23.e215 10851218

[B5] Moody G Mark R Goldberger A PhysioNet: Physiologic signals, time series, and related open source software for basic, clinical, and applied research Engineering in Medicine and Biology Society,EMBC, 2011 Annual International Conference of the IEEE 2011 8327 8330 [http://dx.doi.org/10.1109/IEMBS.2011.6092053] 10.1109/IEMBS.2011.6092053 22256277

[B6] Hug C Clifford GD Reisner AT Clinician blood pressure documentation of stable intensive care patients: an intelligent archiving agent has a higher association with future hypotension Crit Care Med 2011 39 5 1006 1014 [http://journals.lww.com/ccmjournal/Abstract/2011/05000/Clinician_blood_pressure_documentation_of_stable.12.aspx]. [Epub ahead of print] 10.1097/CCM.0b013e31820eab8e 21336136 PMC3102134

[B7] Sun J Reisner A Saeed M Heldt T Mark R The cardiac output from blood pressure algorithms trial Crit Care Med 2009 37 72 80 10.1097/CCM.0b013e3181930174 19112280 PMC3107992

[B8] Li Q Mark RG Clifford GD Robust heart rate estimation from multiple asynchronous noisy sources using signal quality indices and a Kalman Filter IOP Physiol Meas 2008 29 15 32 [http://www.ncbi.nlm.nih.gov/pmc/articles/PMC2259026]. [(Awarded the Martin Black Prize for Best Paper in Physiological Measurement in 2008)] 10.1088/0967-3334/29/1/002 PMC2259026 18175857

[B9] phpPgAdmin phpPgAdmin [http://phppgadmin.sourceforge.net]. [(accessed 20th February 2012)]

[B10] Murphy SN Weber G Mendis M Gainer V Chueh HC Churchill S Kohane I Serving the enterprise and beyond with informatics for integrating biology and the bedside (i2b2) J Am Med Inform Assoc 2010 17 2 124 130 [http://jamia.bmj.com/content/17/2/124.abstract] 10.1136/jamia.2009.000893 20190053 PMC3000779

[B11] Google Google Web Toolkit [http://code.google.com/webtoolkit/]. [(accessed 20th February 2012)]

[B12] Sencha Ext GWT [http://www.sencha.com/products/extgwt/]. [(accessed 20th February 2012)]

[B13] Oracle JDBC [http://docs.oracle.com/javase/7/docs/technotes/guides/jdbc/index.html]. [(accessed 20th February 2012)]

[B14] Le Gall JR Loirat P Alperovitch A Glaser P Granthil C Mathieu D Mercier P Thomas R Villers D A simplified acute physiology score for ICU patients Crit Care Med 1984 12 11 975 977 [http://www.ncbi.nlm.nih.gov/pubmed/6499483] 10.1097/00003246-198411000-00012 6499483

[B15] Chertow GM Burdick E Honour M Bonventre JV Bates DW Acute kidney injury, mortality, length of stay, and costs in hospitalized patients J Am Soc Nephrol 2005 [http://jasn.asnjournals.org/content/early/2005/09/21/ASN.2004090740.short] 10.1681/ASN.2004090740 16177006

[B16] Mehta R Kellum J Shah S Molitoris B Ronco C Warnock D Levin A the Acute Kidney Injury Network Acute kidney injury network: report of an initiative to improve outcomes in acute kidney injury Crit Care 2007 11 2 R31 [http://ccforum.com/content/11/2/R31] 10.1186/cc5713 17331245 PMC2206446

[B17] Mandelbaum T Scott DJ Lee J Mark RG Malhotra A Waikar S Howell MD Talmor DS Outcome of critically ill patients with acute kidney injury using the acute kidney injury network criteria Crit Care Med 2011 39 12 2659 2664 [Preprint available online 14 July 2011] 21765352 10.1097/CCM.0b013e3182281f1b PMC3213281

[B18] Mandelbaum T Lee J Scott DJ Mark RG Malhotra A Howell MD Talmor D Empirical relationships among oliguria, creatinine, mortality, and renal replacement therapy in the critically ill Intensive Care Medin press 10.1007/s00134-012-2767-x PMC3580030 23223822

[B19] Celi L Hinske LC Alterovitz G Szolovits P An artificial intelligence tool to predict fluid requirement in the intensive care unit: a proof-of-concept study Crit Care 2008 12 6 R151 [http://ccforum.com/content/12/6/R151]. [See related commentary by Lane and Boyd. http://ccforum.com/content/13/1/111] 10.1186/cc7140 19046450 PMC2646316

[B20] Moody GB Lehman LH Predicting acute hypotensive episodes: The 10th annual physioNet/computers in cardiology challenge Comput Cardiol 2009 36 541 544 [http://www.cinc.org/Proceedings/2009/pdf/0541.pdf] 20842209 PMC2937253

[B21] Moody GB The PhysioNet/Computing in Cardiology Challenge 2010: Mind the Gap Comput Cardiol 2010 37 305 308 [http://cinc.mit.edu/archives/2010/pdf/0305.pdf] PMC3136865 21766058

[B22] Celi LAG Lee J Scott DJ Panch T Mark RG Collective experience: a database-fuelled, inter-disciplinary team-led learning system J Comput Sci Eng 2012 6 51 59 10.5626/JCSE.2012.6.1.51 PMC3678291 23766887

